# Correlation between Platelet Count and Lung Dysfunction in Multiple Trauma Patients—A Retrospective Cohort Analysis

**DOI:** 10.3390/jcm11051400

**Published:** 2022-03-03

**Authors:** Frederik Greve, Olivia Mair, Ina Aulbach, Peter Biberthaler, Marc Hanschen

**Affiliations:** 1 Department of Trauma Surgery, Klinikum Rechts der Isar, Technical University of Munich, 81675 Munich, Germany; oliviaanna.mair@mri.tum.de (O.M.); ina.aulbach@charite.de (I.A.); peter.biberthaler@mri.tum.de (P.B.); marc.hanschen@mri.tum.de (M.H.); 2 Department of Traumatology and Reconstructive Surgery, Charité-Universitätmedizin Berlin, 12203 Berlin, Germany

**Keywords:** platelets, trauma, immune system, posttraumatic organ failure, posttraumatic lung dysfunction, posttraumatic hyperinflammation

## Abstract

(1) Background: Current findings emphasize the potential contribution of platelets to the immunological response after severe trauma. As clinical relevance remains unclear, this study aims to analyze the correlation between platelets and lung dysfunction in severely injured patients. (2) Methods: We retrospectively enrolled all multiple trauma patients presenting to our level 1 trauma center from 2015 to 2016 with an Injury-Severity Score (ISS) ≥ 16. Apart from demographic data, platelet counts and PaO_2_/FiO_2_ as an approximate indicator for lung physiology were analyzed and correlated on subsequent days after admission. (3) Results: 83 patients with a median ISS of 22 (IQR 18–36) were included. Compared to day 1, platelet counts were decreased on day 3 (*p* ≤ 0.001). Platelet counts were significantly lower on day 3 in patients with an ISS ≥ 35 (*p* = 0.011). There were no differences regarding PaO_2_/FiO_2_ index. Correlation analysis revealed a positive link between increased platelet counts and PaO_2_/FiO_2_ index on day 1 only in severely injured patients (*p* = 0.007). (4) Conclusions: This work supports the concept of platelets modulating the posttraumatic immune response by affecting lung dysfunction in the early phase after multiple trauma in dependence of injury severity. Our findings contribute to the understanding of the impact of platelets on systemic processes in multiple trauma patients.

## 1. Introduction

Trauma-induced injury is the leading cause of death among people until 44 years of age in the United States as well as one of the leading global causes of death and disability [1,2]. Late posttraumatic mortality is caused by systemic hyperinflammation, leading to multiple organ failure (MOF) with high lethality rates up to 50% [3,4,5,6].

Multifactorial pathophysiological mechanisms contribute to increased sensitivity and risk of MOF in the early stages of multiple trauma. The primary components are the pro-inflammatory systemic inflammatory response syndrome (SIRS) and compensatory anti-inflammatory response syndrome (CARS). High intensity and disbalance of these conflicting trauma-induced inflammatory responses trigger the progress of inflammation and development of organ dysfunction and MOF [7,8,9,10,11,12].

Lung injury is frequently observed after multiple trauma and is either caused directly (e.g., thoracic trauma) or indirectly in the context of posttraumatic hyperinflammation or sepsis [13,14,15]. Triggered by SIRS, activated leukocytes migrate into the pulmonary interstitium. Complex intercellular pathways and various cytokines lead to increased endothelial permeability with consecutive alveolar edema and impaired gas exchange. This is followed by a local inflammation, which further contributes to cytokine release and promotes systemic inflammation leading to MOF [16,17,18]. Lung injury clinically manifests as acute respiratory distress syndrome (ARDS), which, according to the latest definition, consists of acute hypoxemia, conspicuous radiological investigations, and exclusion of hydrostatic edema due to cardiac failure [19].

Recently, the impact of platelets on the posttraumatic immune disturbance gained increasing interest. It is well known that platelets serve as immunological mediators besides their distinctive function during hemostasis [20,21,22,23,24,25,26].

Several findings from animal studies indicate that especially in the pathophysiology of lung injury, platelet–neutrophil interactions seem to play a crucial role [27,28,29,30,31]. Driven by pro-inflammatory mediators, platelets adhere to lung capillary endothelial cells, become activated, and release chemokines and lipid mediators [27,28,32,33]. This is followed by activation of attached neutrophils, additional capturing of circulating leukocytes from the blood flow, and further release of pro-inflammatory mediators by endothelial cells [32,34]. Currently, our understanding of the pro- and anti-inflammatory impact of platelets is limited and the subject of ongoing studies.

Several registry studies focused on risk factors for the development of either MOF or ARDS after multiple trauma [35,36,37,38,39]. However, studies reflecting the direct clinical influence of platelets on injury-induced lung impairment are limited. Thus, the present work investigates the correlation between platelet count and lung dysfunction in multiple injury patients. As recent findings revealed a demographic influence on posttraumatic platelet counts [37,40] as well as on MOF and ARDS [35,39,41], we hypothesized that a potential correlation between platelet count and PaO_2_/FiO_2_ index would differ in varying subgroups of gender, age, and injury severity. We aim for a transfer of gaining molecular understanding of platelet interaction to a clinical setting to improve the overall understanding of the immunoinflammatory impact of platelets during posttraumatic hyperinflammation.

## 2. Materials and Methods

The key objective of this study is the clinical investigation of the influence of posttraumatic platelet counts on PaO_2_/FiO_2_ index as approximate measure of pulmonary end-organ failure in multiple trauma patients. For comprehensive evaluation, the posttraumatic dynamics of platelet counts and PaO_2_/FiO_2_ index were additionally analyzed.

We included all multiple trauma patients from our level 1 trauma center in this retrospective analysis from 2015 to 2016. Inclusion was prompted by an injury-severity score (ISS) of 16 or above. The study protocol was approved by the ethics committee of the Technical University of Munich (vote No. 129/17 S). The study was registered in the German Clinical Trial Registry (www.drks.de (accessed on 1 December 2021), trial number: DRKS00027235) and linked to the international Clinical Trials Registry Platform of the World Health Organization (https://trialsearch.who.int (accessed on 1 December 2021)).

### 2.1. Descriptive Analysis

Patient data were analyzed for demographic information (gender, age), cause of injury (blunt vs. penetrating), injury patterns, platelet count (G/l), PaO_2_/FiO_2_ index (mmHg), ICU stay, and outcome (survival vs. non-survival). For assessment of trauma severity, the ISS was calculated as per definition using the Abbreviated Injury Scale (AIS) [42]. This anatomical grading method provides an overall score for patients suffering from multiple injuries. Several body regions are scored from 0–6 (no injury up to non-survivable injury). The top three severity scores are squared and added. The range of the ISS is given from 0–75 [43]. Platelet count and PaO_2_/FiO_2_ index (also called Horovitz quotient or oxygenation index) as approximate measure of posttraumatic lung dysfunction were assessed in the early posttraumatic phase on day 1 and day 3 after admission to our trauma bay. For assessment of dynamic changes, platelet counts and PaO_2_/FiO_2_ index on day 3 were compared to base values on day 1. Statistical testing was performed by use of paired *t*-test after testing for normal distribution (D’Agostino and Pearson test). Testing for differences within the subgroups was performed by *t*-test and Mann–Whitney *U* test. Descriptive analysis was performed by use of mean and standard deviation in case of normal distribution or median and interquartile range in case of non-normally distributed parameters.

### 2.2. Correlation Analysis

As descriptive analysis only allows for interpretation of the kinetics and comparison between the respective subgroups, additional correlation analysis was performed to investigate a potential relationship between platelet count and PaO_2_/FiO_2_ index.

Correlation between PaO_2_/FiO_2_ index and platelet count for the entire study population and for patients in dependence of thoracic trauma—assessed for each day—was performed by use of Pearson (in case of normal distribution and linear relationship) or Spearman’s correlation coefficient. The identic correlation as named above was performed after dividing the study population into three subgroups (gender: male/female; ISS: <35/≥35; age: <60 years/≥60 years). Correlation coefficients of PaO_2_/FiO_2_ index and platelet count of the respective subgroups were compared. A *Z*-test was performed to compare correlation coefficients from independent samples. For application of the *Z*-test, Fisher’s *Z*-transformation was considered.

The level of significance was set as *p* < 0.05. Statistical testing was performed by use of GraphPad PRISM Software (San Diego, CA, USA).

## 3. Results

### 3.1. Descriptive Data

For this study, data from 189 patients hospitalized to our trauma center were screened. With an ISS higher than 16 and full datasets for the measurement of PaO_2_/FiO_2_ index and platelet count on subsequent days, 83 individuals were found to be appropriate for inclusion in this research.

The leading cause of injury was a blunt trauma mechanism. The largest proportion was diagnosed with head/neck or extremities/pelvic trauma. Almost half of the patients presented thoracic trauma. The majority suffered a severe trauma expressed by a median ISS of 22 (IQR 18–36). The majority of the patients were admitted to ICU with an average stay of 8 days. Lethal outcome was observed in 10% of the cases. For further details, please see Table 1.

### 3.2. Dynamics of Platelet Counts and PaO_2_/FiO_2_ Index

**Figure 1:**
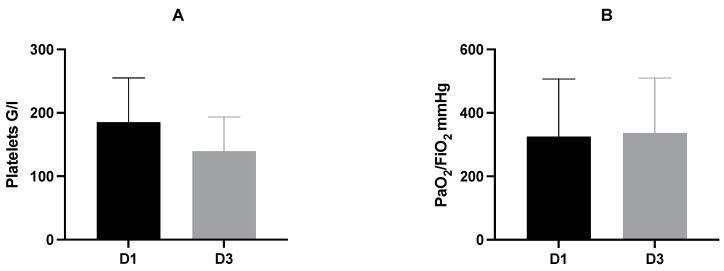
Mean and standard deviation of platelet count (**A**) and PaO2/FiO2 index (**B**) on D1 and D3 after trauma. Mean platelet count on D3 was significantly decreased compared to D1 (D1: mean 185.5 ± 69.7 G/l vs. D3: mean 139.9 ± 53.5 G/l; *p* ≤ 0.001). There was no significant difference of the PaO_2_/FiO_2_ index between D1 and D3 (D1: mean 325 ± 181.6 mmHg vs. D3: mean 336.8 ± 172.8 mmHg; *p* = 0.755). D, day.

Platelet counts were gathered in the early phase after admission and analyzed for differences between day 1 and day 3 after trauma (Table 2, Figure 1).

In relation to day 1 as base value, platelet counts decreased significantly on day 3 (D1: mean 185.5 G/l ± 69.6 G/l vs. D3: mean 139.9 G/l ± 53.5 G/l; *p* ≤ 0.001).

**Figure 2:**
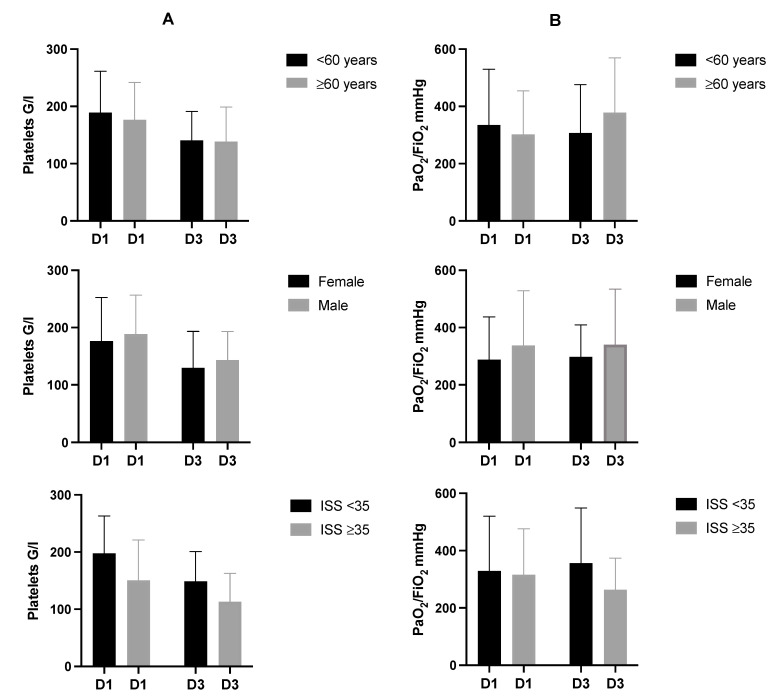
Descriptive subgroup analysis according to age, gender, and injury severity. Mean and standard deviation of platelet count (**A**) and PaO_2_/FiO_2_ index (**B**) on D1 and D3 after trauma. Comparison of platelet count and PaO_2_/FiO_2_, index in dependence of age and gender did not show any significant difference. Platelet count was significantly increased in patients with less severe injury (ISS < 35) compared to severely injured patients (ISS ≥ 35) on D1 (ISS < 35: mean 197.7 ± 65.5 G/l vs. ISS ≥ 35: mean 150.6 ± 70.9 G/l; *p* = 0.012) and D3 (ISS < 35: mean 149.0 ± 52.2 G/l vs. ISS ≥ 35: mean 113.2 ± 49.4 G/l; *p* = 0.011). There was no significant difference regarding PaO_2_/FiO_2_ index. D, day.

Platelet counts were also analyzed within subgroups in dependence of age, gender, and injury severity (Table 3, Figure 2). Age and gender did not affect platelet counts on day 1 and day 3 after trauma. No significant differences between patients younger/older than 60 years and male/female patients were detected on day 1 and day 3 after admission for multiple trauma. In severely injured patients (ISS ≥ 35), platelet counts were significantly decreased on day 1 (ISS < 35: mean 197.7 ± 65.5 G/l vs. ISS ≥ 35: mean 150.6 ± 70.9 G/l; *p* = 0.012 *) and day 3 (ISS < 35: mean 149.0 ± 52.2 G/l vs. ISS ≥ 35: mean 113.2 ± 49.4 G/l; *p* = 0.011 *).

In analogy to platelet counts, PaO_2_/FiO_2_ index was determined on day 1 and day 3 after admission (Table 2, Figure 1) and additionally analyzed within the subgroups (Table 4, Figure 2). There were no significant differences.

### 3.3. Correlation Analysis

**Figure 3:**
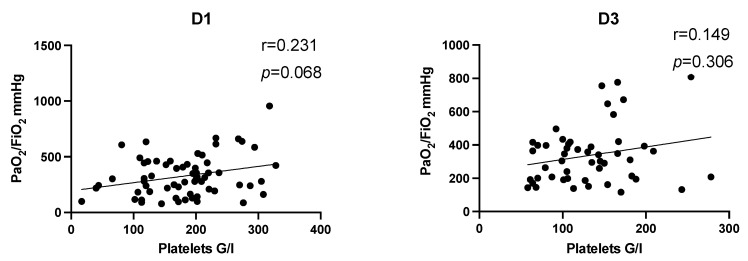
Correlation analysis of the entire study population on D1 and D3 after multiple trauma. Correlation analysis showed a slight positive correlation of platelet count and PaO_2_/FiO_2_ without reaching level of significance. The effect tends to be more pronounced on D1 (r = 0.231, *p* = 0.068). D, day; r, Spearman/Pearson correlation coefficient; *p*, level of significance.

In a first step, correlation analysis of the entire study population was performed on day 1 and day 3 after multiple trauma (Figure 3). Correlation coefficients revealed a trend that increased platelet counts tend to be associated with increased PaO_2_/FiO_2_ index without reaching level of significance (D1: r = 0.23, *p* = 0.068; D3: r = 0.149, *p* = 0.306). To account for the potential impact of thoracic trauma, we performed correlation analysis after dividing the study population in dependence of diagnosed thoracic trauma. There was no significant correlation in patients with or without thoracic trauma between platelet count and PaO_2_/FiO_2_ index on day 1 (thoracic trauma: r = 0.19, *p* = 0.258; no thoracic trauma: r = 0.32, *p* = 0.109) and day 3 (thoracic trauma: r = 0.07, *p* = 0.723; no thoracic trauma: r = 0.287, *p* = 0.248).

The same analysis was performed in the respective subgroups to account for a potential influence of age, gender, and injury severity.

**Figure 4:**
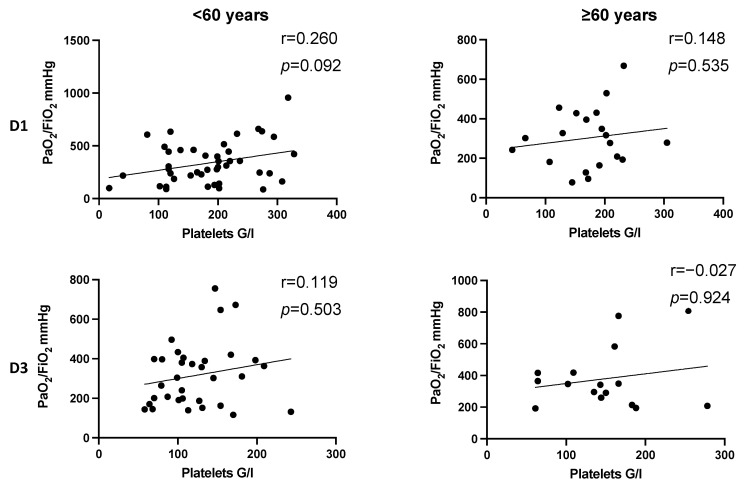
Correlation between platelet count and PaO_2_/FiO_2_ index following injury in dependence of age. There is no significant correlation between platelet count and PaO_2_/FiO_2_ index on D1 and D3 in dependence of age after trauma. Correlation coefficients show a slight positive correlation of platelet counts and PaO_2_/FiO_2_ index in younger and older patients. In younger patients, the effect seems to be more pronounced (D1: r = 0.26, *p* = 0.092; D3: r = 0.119, *p* = 0.503). D, day; r, Spearman/Pearson correlation coefficient; *p*, level of significance.

In patients younger than 60 years, a trend was detected that increased platelet counts seem to correlate with high PaO_2_/FiO_2_ index on day 1 after multiple trauma (r = 0.263, *p* = 0.092) (Figure 4, D1). The observations did not reach level of significance.

**Figure 5:**
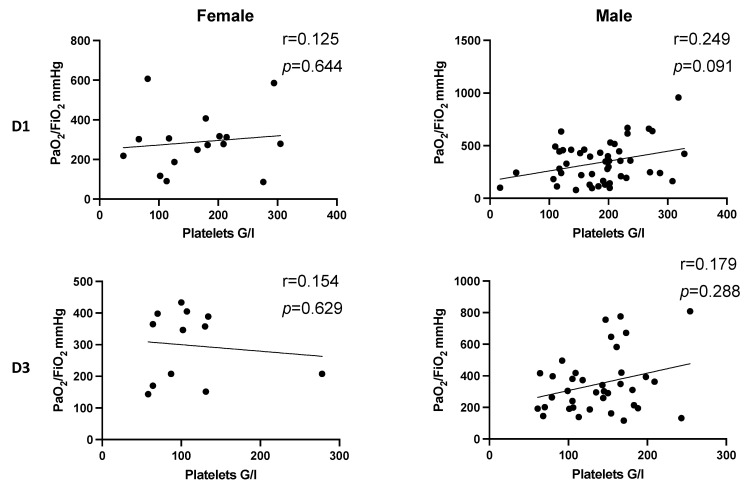
Correlation between platelet count and PaO_2_/FiO_2_ index following injury in dependence of gender. There is no significant correlation between platelet count and PaO_2_/FiO_2_ index on D1 and D3 in dependence of gender after trauma. Correlation coefficients in male patients on D1 (r = 0.249, *p* = 0.091) and D3 (r = 0.179, *p* = 0.288) indicate a pronounced effect that increased platelet counts tend to be associated with higher values of PaO_2_/FiO_2_ index. D, day; r, Spearman/Pearson correlation coefficient; *p*, level of significance.

In dependence of gender, there also was a pronounced positive correlation in male patients on day 1 after multiple trauma (D1: r = 0.249, *p* = 0.091) (Figure 5, D1). However, a significant correlation was not observed.

**Figure 6:**
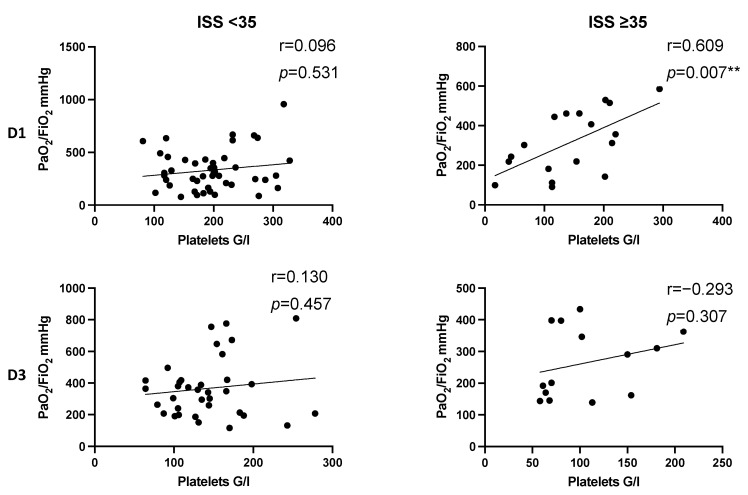
Correlation between platelet count and PaO_2_/FiO_2_ index following injury in dependence of injury severity (ISS). Correlation coefficients reveal a significantly positive correlation on D1 in severely injured patients (r = 0.609, *p* = 0.007). Increased platelet count seems to be associated with higher values of PaO_2_/FiO_2_ index. On D3, there is a pronounced positive correlation in severely injured patients without reaching level of significance. (r = 0.293, *p* = 0.307). D, day; r, Spearman/Pearson correlation coefficient; *p*, level of significance.

In severely injured patients presenting an ISS ≥ 35, we detected a significant positive correlation indicating that increased platelet counts might correlate with increased PaO_2_/FiO_2_ index on day 1 after multiple trauma (D1: r = 0.609, *p* = 0.007 **). The effect was neither present in less severely injured patients (ISS < 35 D1: r = 0.096, *p* = 0.531; D3: r = 0.13, *p* = 0.457), nor did it last until day 3 (r = 0.293, *p* = 0.307) (Figure 6).

Subgroup analysis, which took into account the effects of age, gender, and injury severity, revealed various association patterns as shown in Figure 2, Figure 3, Figure 4, Figure 5 and Figure 6. The level of significance was calculated as part of the statistical workup as shown in the legends of Figure 2, Figure 3, Figure 4, Figure 5 and Figure 6. Table 5 allows for direct comparison of the given correlations, including the detailed indication of the patients included for each subgroup. For statistical comparison of the correlation coefficients, the *Z*-test was utilized.

## 4. Discussion

In the setting of posttraumatic hyperinflammation, activated leukocytes migrate into the pulmonary microcirculation, leading to endothelial permeability and tissue edema formation with consecutive impaired gas exchange in the pulmonary alveoli [16,17,18]. Release of inflammatory mediators from the injured lung further contributes to additional end organ damage. Therefore, lung injury is an obligate early step and pacemaker in the development of MOF [38].

The assumption that platelets merely are a key player in hemostasis is outdated. Platelets contribute to inflammation as they release granules with pro-inflammatory cytokines, interact with neutrophils, and amplify endothelial-mediated inflammation and tissue injury [34,44].

In this study, our aim was to transfer increasing molecular understanding to a clinical setting. We performed a descriptive and correlation analysis of 83 multiple trauma patients (median ISS 22, IQR 18–36) to investigate the direct influence of platelet counts on PaO_2_/FiO_2_ index as a parameter for lung dysfunction.

### 4.1. Descriptive Analysis

#### Dynamics of Platelet Counts and PaO_2_/FiO_2_ Index after Multiple Trauma

Facing the entire study population, we detected a significant decrease of platelets on day 3 compared to day 1. There was no difference of PaO_2_/FiO_2_ index within the first days after trauma (Figure 1, Table 2). Subgroup analysis revealed significantly decreased platelet counts on day 1 in severely injured patients compared to less severely injured patients. There were no differences regarding PaO_2_/FiO_2_ index in dependence of age, gender, and injury severity (Figure 2, Table 3 and Table 4).

Thrombocytopenia in the initial phase after trauma was also detected by Nydam and coworkers. In line with our results, low platelet counts were associated with increased injury severity. In addition, they were able to identify low post-injury platelet counts as a major independent risk factor for MOF and death, whereas higher platelet counts showed a protective effect several days after trauma [37]. Hefele and coworkers investigated post-injury dynamics of platelet counts and described low concentrations in the early phase and a recovery around ten days after multiple trauma with impaired function in thrombelastometry. Analogous to our findings, they detected decreased platelet counts in severely injured patients [40].

Thrombocytopenia in the early critical phase might be caused by dilution effects due to high-volume substitution in heavily injured patients. Furthermore, thrombocytopenia is associated with platelet sequestration in damaged pulmonary tissue by interactions with neutrophils promoted by adhesion molecule P-selectin [27,30,45,46]. We were surprised not to detect any differences in dynamics of PaO_2_/FiO_2_ index within the first 72 h after trauma. Several studies identified the lung to be the first affected organ in the cascade of MOF [13,14,15,38]. The large pulmonary capillary system filters the entire cardiac output, which is loaded with cytokines and inflammatory cells, such as neutrophils and macrophages. Ciesla and coworkers investigated a large multiple trauma collective for characterization of the onset of respective organ system impairment in the development of MOF. They detected lung dysfunction with decreased PaO_2_/FiO_2_ index to occur 1.6 days after trauma, followed by heart, liver, and kidney failure. The severity of lung dysfunction correlated with the extent of damage to other organ systems [38]. According to Sauaia and coworkers, almost every severe multiple trauma patient develops lung dysfunction, with the lowest influence on mortality. Mortality is believed to be highest for cardiovascular dysfunction, followed by acute kidney failure, liver injury, and lung dysfunction [7,47]. As several studies describe that age, gender, and injury severity influence MOF and lung dysfunction, we would have expected differences in the respected subgroups regarding PaO_2_/FiO_2_ index [7,35,36].

### 4.2. Correlation between Platelet Count and PaO_2_/FiO_2_ Index

We did not detect a significant correlation between platelet count and PaO_2_/FiO_2_ index facing the entire study population after multiple trauma (Figure 3). Correlation analysis within the subgroups showed no effect for age and gender (Figure 4 and Figure 5, Table 5), but we detected a significantly positive correlation for severely injured patients on day 1 after multiple trauma. On day 3, there still is a tendency without reaching level of significance (Figure 6, Table 5). This indicates that decreased platelet count could be associated with decreased PaO_2_/FiO_2_ index as a parameter for impaired lung physiology within 48 h after severe multiple trauma (ISS ≥ 35).

We hypothesize that low platelet counts are caused by sequestration into the lungs as mentioned above [30]. This is followed by local inflammation leading to epithelial damage and fluid infiltration, which reduces alveolar integrity with dysfunctional gas exchange as a consequence [48,49,50]. High platelet counts might predict a less severe posttraumatic course.

Platelets are involved in the pathophysiology of acute lung injury merely by recruitment of neutrophils [34]. After activation, platelets release the content of their granules (procoagulant and fibrinolytic factors, pyrophosphate, calcium, and adhesion molecules) undergo change of shape and upregulate the expression of adhesion molecules (P-selectin, PECAM-1, Glycoprotein IIb/IIIa, fibronectin, and thrombospondin) [34,44,51]. Platelet attachment to pulmonary capillary endothelium is mainly mediated by P-selectin [28]. Subsequent attachment of neutrophils then leads to platelet–neutrophil interactions inducing local tissue injury [34]. Blockade of P-selectin in a rodent animal model for acute lung injury showed superior outcomes due to reduced platelet–neutrophil aggregates making this a potential therapeutic target [27].

Kasotakis and coworkers investigated the effect of platelet transfusions in multiple trauma patients. According to their results, high-volume platelet transfusions are associated with the development of ARDS [52]. This supports the theory of a detrimental effect of platelets on lung physiology. In contradiction to their findings, we detected that increasing platelet counts (without supplementation) are associated with improved lung function. The impact of transfused platelet units could potentially be amplified by HLA antibodies responsible for transfusion-related acute lung injury (TRALI) [52].

Several studies additionally reported that prehospital antiplatelet therapy was associated with lower incidence of lung dysfunction [53,54]. However, a large prospective, randomized, placebo-controlled clinical trial investigating the effect of aspirin on the development of ARDS in patients at risk ruled out a potential benefit [55]. Recent results from a multiple trauma animal model showed promising therapeutic results by use of tranexamic acid as an additional example of potential involvement of the coagulation system in lung dysfunction development. After administration of tranexamic acid, Wu and coworkers detected a decreased pulmonary platelet–neutrophil infiltration with reduced edema formation by increased integrity of epithelial barrier function [56].

In summary, our findings are in line with the existing literature and contribute to the understanding that platelets are involved in the pathophysiology of posttraumatic lung dysfunction. Precise pathways are still not understood and remain to be elucidated.

### 4.3. Limitations

The study’s design, data processing, and data interpretation imply limitations that must be taken into account. The retrospective character of the study limits the control of data quality and data completeness although all efforts were made to ensure for best possible accuracy.

PaO_2_/FiO_2_ index is frequently used to sufficiently describe hypoxemia and lung dysfunction [37,38,52]. However, the current definition of ARDS additionally requires chest radiographs and assessment of right heart function, which are not routinely assessed in our clinic [19].

In addition, injury patterns (e.g., severity of thoracic trauma) and individual therapy (e.g., platelet transfusions) of multiple trauma patients vary significantly. Therefore, the heterogeneity of the patient population has the potential to bias the results. We are aware that direct thoracic trauma in severely injured individuals might have an impact on early PaO_2_/FiO_2_ index and potentially influences our results. However, there was no difference for the entire study population in dependence of thoracic trauma. Due to the rather small sample size, we waived additional subgroup analysis in dependence of thoracic trauma. Unfortunately, we were unable to assess platelet transfusions in patients’ medical records, which could further bias our results due to the association with ARDS in multiple trauma patients [52]. We further point out that a study population of 83 individuals is underpowered to derive clear clinical conclusions yet sufficient enough for findings as basis for future studies with larger data sets (e.g., registry studies including the impact of direct thoracic trauma).

Finally, all attempts have been made to discuss the results of the present study with the highest degree of caution. Strong correlation, even reaching level of significance, does not imply causation. Therefore, further studies are needed to support our results.

## 5. Conclusions

In conclusion, the present retrospective clinical study is the first to investigate the clinical relevance of posttraumatic platelet count, highlighting the correlation between platelets and injury-induced respiratory organ failure.

We were able to present alterations in the dynamics of posttraumatic platelet counts. Severity of injury seems to have a pronounced impact within the first 72 h after trauma, whereas gender and age did not present a modulating influence. Dynamics of PaO_2_/FiO_2_ index as a parameter for lung injury remained stable without being influenced by gender, age, or trauma severity. Correlation analysis revealed that low platelet counts tend to be associated with impaired lung physiology only in severely injured patients. Being aware that correlation does not imply causation, our data resonate with previous findings supporting the theory that platelets tend to contribute to the development of posttraumatic lung dysfunction. Our clinical data cannot determine the exact mechanisms but can add further knowledge to the overall understanding of the complex posttraumatic immunological processes and build a basis for future studies.

## Figures and Tables

**Table 1:** Demographic patient data of the study population.

Demography	Number *n* (%)	Median (IQR Q_25_–Q_75_)/Mean ± SD
Included patients	83 (100%)	-
Age		Median 51 years (34–64 years)
<60 years	57 (68.7%)	Median 43 years (29–51 years)
≥60 years	26 (31.3%)	Median 73 years (66–76 years)
Gender		
Male	62 (74.7%)	-
Female	21 (25.3%)	-
Mechanisms of injury		
Blunt trauma	76 (91.6%)	-
Penetrating trauma	7 (8.4%)	-
AIS		
Head/neck	59 (71.1%)	Median 3 (0–4)
Face	28 (33.7%)	Median 0 (0–2)
Thorax	45 (54.2%)	Median 2 (0–3)
Abdomen	28 (33.7%)	Median 0 (0–3)
Extremities/pelvis	62 (74.7%)	Median 3 (0–4)
Other	25 (30.1%)	Median 0 (0–1)
ISS	-	Median 22 (18–36)
<35	62 (74.7%)	Median 19 (17–25)
≥35	21 (25.3%)	Median 41 (38–57)
ICU stay		
Days on ICU	1–56 days	Mean 8.3 ± 13.0 days
Patients without ICU stay	16 (19%)	Mean 15.1 ± 5.8 days
Patients with ICU stay	67 (81%)	Mean 10.1 ± 13.7 days
Mortality		
Deaths	10 (12%)	-
Survivors	73 (88%)	-

SD, standard deviation; IQR, interquartile range; AIS, abbreviated injury scale; ISS, injury-severity score; ICU, intensive care unit.

**Table 2:** Platelet counts and PaO_2_/FiO_2_ on day (D) 1 and 3 of the entire study population.

	Platelet Count (G/L) (Mean ± SD)	*p*	PaO_2_/FiO_2_ (mmHg) (Mean ± SD)	*p*
D1	185.5 ± 69.6	-	325 ± 181.6	
D3	139.9 ± 53.5	≤0.001 ***	336.8 ± 172.8	0.755

Testing for statistical significance was performed between D3 and D1 as base value during admission in the trauma bay. SD, standard deviation; *** = *p* ≤ 0.001; level of significance was set as *p* < 0.05.

**Table 3:** Platelet counts on day (D) 1 and 3 in dependence of age, gender and injury severity.

Platelet Count (G/l)	<60 Years (Mean ± SD)	≥60 Years (Mean ± SD)	*p*
D1	189.4 ± 71.7	176.7 ± 65.3	0.437
D3	140.5 ± 50.5	138.7 ± 60.2	0.895
	**Female**	**Male**	***p***
D1	176.4 ± 76.0	188.6 ± 67.7	0.518
D3	129.8 ± 63.6	143.3 ± 49.8	0.408
	**ISS < 35**	**ISS ≥ 35**	***p***
D1	197.7 ± 65.5	150.6 ± 70.9	0.012 *
D3	149.0 ± 52.2	113.2 ± 49.4	0.011 *

SD, standard deviation; ISS, injury-severity score; * = *p* < 0.05; level of significance was set as *p* < 0.05.

**Table 4:** PaO_2_/FiO_2_ index on day (D) 1 and 3 in dependence of age, gender, and injury severity.

PaO_2_/FiO_2_ Index (mmHg)	<60 Years (Mean ± SD)	≥60 Years (Mean ± SD)	*p*
D1	353.3 ± 194.6	302.8 ± 151.3	0.672
D3	307.3 ± 169.1	378.5 ± 190.7	0.236
	**Female**	**Male**	***p***
D1	288.4 ± 148.8	337.4 ± 191.1	0.411
D3	297.9 ± 114.4	340.2 ± 193.9	0.881
	**ISS < 35**	**ISS ≥ 35**	***p***
D1	328.9 ± 190.7	315.5 ± 160.7	>0.999
D3	355.8 ± 192.9	263.8 ± 109.8	0.117

SD, standard deviation; ISS, injury-severity score; level of significance was set as *p* < 0.05.

**Table 5:** Correlation coefficients between platelet count and PaO_2_/FiO_2_ index in dependence of age, gender, and injury severity and comparison between correlation coefficients (*Z*-test).

Age	<60				≥60				Comparison of Correlation Coefficients	
	r	*p*		*n*	r	*p*		*n*	*Z*-test	*p*
D1	0.260	0.092	ns	43	0.148	0.535	ns	20	0.404	0.686
D3	0.119	0.503	ns	33	−0.027	0.924	ns	16	0.441	0.658
**Gender**	**Female**				**Male**				**Comparison of Correlation Coefficients**	
	**r**	***p***		***n***	**r**	***p***		***n***	***Z*-test**	***p***
D1	0.125	0.644	ns	16	0.249	0.091	ns	47	–0.408	0.684
D3	0.154	0.629	ns	12	0.179	0.288	ns	37	–0.069	0.946
**ISS**	**<35**				**≥35**				**Comparison of Correlation Coefficients**	
	**r**	***p***		***n***	**r**	***p***		***n***	***Z*-test**	***p***
D1	0.096	0.531	ns	45	0.609	0.007	**	18	–2.031	0.042 *
D3	0.130	0.457	ns	35	−0.293	0.307	ns	14	1.238	0.216

r, Spearman/Pearson correlation coefficient; *p*, level of significance; *n*, number of correlated pairs; ns, not significant; * *p* < 0.05; ** *p* < 0.01; level of significance was set as *p* < 0.05.

## Data Availability

The data presented in this study are available on request from the corresponding author. The data are not publicly available due to retrospective data collection without the necessity for informed consent (see above).

## References

[1] Heron M. (2021). Deaths: Leading Causes for 2019. Natl. Vital Stat. Rep..

[2] World Health Organization The Top 10 Causes of Death. https://www.who.int/news-room/fact-sheets/detail/the-top-10-causes-of-death.

[3] Demetriades D., Kimbrell B., Salim A., Velmahos G., Rhee P., Preston C., Gruzinski G., Chan L. (2005). Trauma Deaths in a Mature Urban Trauma System: Is “Trimodal” Distribution a Valid Concept?. J. Am. Coll. Surg..

[4] Ciesla D.J., Moore E.E., Johnson J.L., Burch J.M., Cothren C.C., Sauaia A. (2005). A 12-Year Prospective Study of Postinjury Multiple Organ Failure. Arch. Surg..

[5] Cohen J. (2002). The immunopathogenesis of sepsis. Nature.

[6] Brun-Buisson C. (2000). The epidemiology of the systemic inflammatory response. Intensive Care Med..

[7] Sauaia A., Moore F.A., Moore E.E. (2017). Postinjury Inflammation and Organ Dysfunction. Crit. Care Clin..

[8] Zedler S., Faist E. (2006). The impact of endogenous triggers on trauma-associated inflammation. Curr. Opin. Crit. Care.

[9] Hirsiger S., Simmen H.-P., Werner C.M.L., Wanner G.A., Rittirsch D. (2012). Danger Signals Activating the Immune Response after Trauma. Mediat. Inflamm..

[10] Bergmann C.B., Beckmann N., Salyer C.E., Hanschen M., Crisologo P.A., Caldwell C.C. (2021). Potential Targets to Mitigate Trauma- or Sepsis-Induced Immune Suppression. Front. Immunol..

[11] Gentile L.F., Cuenca A.G., Efron P.A., Ang D., Bihorac A., McKinley B.A., Moldawer L.L., Moore F.A. (2012). Persistent inflammation and immunosuppression. J. Trauma Acute Care Surg..

[12] Moore F.A., Moore E.E. (2009). The Evolving Rationale for Early Enteral Nutrition Based on Paradigms of Multiple Organ Failure: A Personal Journey. Nutr. Clin. Pract..

[13] Fry D.E., Pearlstein L., Fulton R.L., Polk H.C. (1980). Multiple System Organ Failure. Arch. Surg..

[14] Faist E., Baue A.E., Dittmer H., Heberer G. (1983). Multiple Organ Failure in Polytrauma Patients. J. Trauma Inj. Infect. Crit. Care.

[15] Regel G., Grotz M., Weltner T., Sturm J.A., Tscherne H. (1996). Pattern of Organ Failure following Severe Trauma. World J. Surg..

[16] Weinacker A.B., Vaszar L.T. (2001). Acute Respiratory Distress Syndrome: Physiology and New Management Strategies. Annu. Rev. Med..

[17] Tasaka S., Hasegawa N., Ishizaka A. (2002). Pharmacology of Acute Lung Injury. Pulm. Pharmacol. Ther..

[18] Bhatia M., Moochhala S. (2004). Role of inflammatory mediators in the pathophysiology of acute respiratory distress syndrome. J. Pathol..

[19] Ranieri V.M., Rubenfeld G.D., Thompson B.T., Ferguson N.D., Caldwell E., Fan E., Camporota L., Slutsky A.S., ARDS Definition of Task Force (2012). Acute Respiratory Distress Syndrome: The Berlin Definition. JAMA.

[20] Elzey B.D., Sprague D.L., Ratliff T.L. (2005). The emerging role of platelets in adaptive immunity. Cell. Immunol..

[21] Clark S.R., Ma A.C., Tavener S.A., McDonald B., Goodarzi Z., Kelly M.M., Patel K.D., Chakrabarti S., McAvoy E., Sinclair G.D. (2007). Platelet TLR4 activates neutrophil extracellular traps to ensnare bacteria in septic blood. Nat. Med..

[22] Henn V., Slupsky J.R., Gräfe M., Anagnostopoulos I., Forster R., Müller-Berghaus G., Kroczek R.A. (1998). CD40 ligand on activated platelets triggers an inflammatory reaction of endothelial cells. Nature.

[23] Danese S., De La Motte C., Reyes B.M.R., Sans M., Levine A.D., Fiocchi C. (2004). Cutting Edge: T Cells Trigger CD40-Dependent Platelet Activation and Granular RANTES Release: A Novel Pathway for Immune Response Amplification. J. Immunol..

[24] Hilf N., Singh-Jasuja H., Schwarzmaier P., Gouttefangeas C., Rammensee H.-G., Schild H. (2002). Human platelets express heat shock protein receptors and regulate dendritic cell maturation. Blood.

[25] Klinger M.H., Jelkmann W. (2002). Review: Role of Blood Platelets in Infection and Inflammation. J. Interf. Cytokine Res..

[26] Engelmann B., Massberg S. (2013). Thrombosis as an intravascular effector of innate immunity. Nat. Rev. Immunol..

[27] Zarbock A., Singbartl K., Ley K. (2006). Complete reversal of acid-induced acute lung injury by blocking of platelet-neutrophil aggregation. J. Clin. Investig..

[28] Kiefmann R., Heckel K., Schenkat S., Dörger M., Węsierska-Gądek J., Goetz A.E. (2004). Platelet-endothelial cell interaction in pulmonary microcirculation: The role of PARS. Thromb. Haemost..

[29] Asaduzzaman M., Lavasani S., Rahman M., Zhang S., Braun O.Ö., Jeppsson B., Thorlacius H. (2009). Platelets support pulmonary recruitment of neutrophils in abdominal sepsis. Crit. Care Med..

[30] Looney M.R., Nguyen J.X., Hu Y., van Ziffle J.A., Lowell C.A., Matthay M.A. (2009). Platelet depletion and aspirin treatment protect mice in a two-event model of transfusion-related acute lung injury. J. Clin. Investig..

[31] Rahman M., Zhang S., Chew M., Ersson A., Jeppsson B., Thorlacius H. (2009). Platelet-Derived CD40L (CD154) Mediates Neutrophil Upregulation of Mac-1 and Recruitment in Septic Lung Injury. Ann. Surg..

[32] Schulz C., Schäfer A., Stolla M., Kerstan S., Lorenz M., Von Brühl M.-L., Schiemann M., Bauersachs J., Gloe T., Busch D.H. (2007). Chemokine Fractalkine Mediates Leukocyte Recruitment to Inflammatory Endothelial Cells in Flowing Whole Blood. Circulation.

[33] Zhu J., Carman C.V., Kim M., Shimaoka M., Springer T.A., Luo B.-H. (2007). Requirement of α and β subunit transmembrane helix separation for integrin outside-in signaling. Blood.

[34] Zarbock A. (2009). The role of platelets in acute lung injury (ALI). Front. Biosci..

[35] Sauaia A., Moore E.E., Johnson J.L., Chin T.L., Banerjee A., Sperry J.L., Maier R.V., Burlew C.C. (2014). Temporal trends of postinjury multiple-organ failure. J. Trauma Acute Care Surg..

[36] Howard B.M., Kornblith L.Z., Hendrickson C.M., Redick B.J., Conroy A.S., Nelson M.F., Callcut A.R., Calfee C.S., Cohen M.J. (2015). Differences in degree, differences in kind. J. Trauma Acute Care Surg..

[37] Nydam T.L., Kashuk J.L., Moore E.E., Johnson J.L., Burlew C.C., Biffl W.L., Barnett C.C., Sauaia A. (2011). Refractory Postinjury Thrombocytopenia Is Associated With Multiple Organ Failure and Adverse Outcomes. J. Trauma Inj. Infect. Crit. Care.

[38] Ciesla D.J., Moore E.E., Johnson J.L., Burch J.M., Cothren C.C., Sauaia A. (2005). The role of the lung in postinjury multiple organ failure. Surgery.

[39] Fröhlich M., Lefering R., Probst C., Paffrath T., Schneider M.M., Maegele M., Sakka S.G., Bouillon B., Wafaisade A. (2014). Epidemiology and risk factors of multiple-organ failure after multiple trauma. J. Trauma Acute Care Surg..

[40] Hefele F., Ditsch A., Krysiak N., Caldwell C.C., Biberthaler P., van Griensven M., Huber-Wagner S., Hanschen M. (2019). Trauma Induces Interleukin-17A Expression on Th17 Cells and CD4+ Regulatory T Cells as Well as Platelet Dysfunction. Front. Immunol..

[41] Sauaia A., Moore F.A., Moore E.E., Haenel J.B., Read R.A., Lezotte D.C. (1994). Early Predictors of Postinjury Multiple Organ Failure. Arch. Surg..

[42] Gennarelli T.A., Woodzin E. (2016). Abbreviated Injury Scale 2005: Update 2008.

[43] Baker S.P., O’Neill B., Haddon W., Long W.B. (1974). The injury severity score: A method for describing patients with multiple injuries and evaluating emergency care. J. Trauma.

[44] Zarbock A., Polanowska-Grabowska R., Ley K. (2007). Platelet-neutrophil-interactions: Linking hemostasis and inflammation. Blood Rev..

[45] Botha A.J., Moore A.F., Moore E.E., Kim F.J., Banerjee A., Peterson V.M. (1995). Postinjury neutrophil priming and activation: An early vulnerable window. Surgery.

[46] Laschke M.W., Dold S., Menger M.D., Jeppsson B., Thorlacius H. (2008). Platelet-dependent accumulation of leukocytes in sinusoids mediates hepatocellular damage in bile duct ligation-induced cholestasis. J. Cereb. Blood Flow Metab..

[47] Ciesla D.J., Moore E.E., Johnson J.L., Cothren C.C., Banerjee A., Burch J.M., Sauaia A. (2006). Decreased progression of postinjury lung dysfunction to the acute respiratory distress syndrome and multiple organ failure. Surgery.

[48] Pugin J., Verghese G., Widmer M.-C., Matthay M.A. (1999). The alveolar space is the site of intense inflammatory and profibrotic reactions in the early phase of acute respiratory distress syndrome. Crit. Care Med..

[49] Ware L.B., Matthay M.A. (2000). The Acute Respiratory Distress Syndrome. N. Engl. J. Med..

[50] Wiener-Kronish J.P., Albertine K.H., Matthay A.M. (1991). Differential responses of the endothelial and epithelial barriers of the lung in sheep to Escherichia coli endotoxin. J. Clin. Investig..

[51] Rendu F., Brohard-Bohn B. (2001). The platelet release reaction: Granules’ constituents, secretion and functions. Platelets.

[52] Kasotakis G., Starr N., Nelson E., Sarkar B., Burke P.A., Remick D.G., Tompkins R.G., The Inflammation and Host Response to Injury Investigators (2019). Platelet transfusion increases risk for acute respiratory distress syndrome in non-massively transfused blunt trauma patients. Eur. J. Trauma Emerg. Surg..

[53] Chen W., Janz D.R., Bastarache J.A., May A.K., O’Neal H.R., Bernard G.R., Ware L.B. (2015). Prehospital Aspirin Use Is Associated With Reduced Risk of Acute Respiratory Distress Syndrome in Critically Ill Patients. Crit. Care Med..

[54] Harr J., Moore E.E., Johnson A.J., Chin T.L., Wohlauer M.V., Maier R.V., Cuschieri J., Sperry J.L., Banerjee A., Silliman C.C. (2013). Antiplatelet Therapy Is Associated with Decreased Transfusion-Associated Risk of Lung Dysfunction, Multiple Organ Failure, and Mortality in Trauma Patients. Crit. Care Med..

[55] Kor D.J., Carter R.E., Park P.K., Festic E., Banner-Goodspeed V., Hinds R., Talmor D., Gajic O., Ware L.B., Gong M.N. (2016). Effect of Aspirin on Development of ARDS in At-Risk Patients Presenting to the Emergency Department. JAMA.

[56] Wu X., Dubick M.A., Schwacha M.G., Cap A.P., Darlington D.N. (2017). Tranexamic Acid Attenuates The Loss of Lung Barrier Function in a Rat Model of Polytrauma and Hemorrhage with Resuscitation. Shock.

